# 1-(Anthracen-1-yl)pyrrolidine-2,5-dione

**DOI:** 10.1107/S160053681201536X

**Published:** 2012-04-21

**Authors:** Sanaz Khorasani, Manuel A. Fernandes

**Affiliations:** a Molecular Sciences Institute, School of Chemistry, University of the Witwatersrand, PO Wits 2050, Johannesburg, South Africa

## Abstract

In the mol­ecular structure of title compound, C_18_H_13_NO_2_, the succinimide ring is orientated away from the plane of the anthracene moiety by 71.94 (4)°. The crystal structure features three different types of inter­molecular inter­actions, *viz.* C—H⋯O, C—H⋯π and π–π bonds. Mol­ecules along the *b* axis stack on each other as a result of π–π inter­actions which have a centroid–centroid distance of 3.6780 (15) Å, while C—H⋯π inter­actions are present between neigbouring stacks. Also, acting between the stacks are the C—H⋯O inter­actions between the aromatic H atoms of the anthracene and the O atoms of the succinimide.

## Related literature

For studies of regio- and sterio-selectivity of substituted anthracenes in Diels–Alder reactions, see: Singh & Ningombom (2010 ▶); Alston *et al.* (1979 ▶); Meek *et al.* (1960 ▶); Kaplan & Conroy (1963 ▶); Verma & Singh (1977 ▶). For a study involving NMR experiments, see: Hubbard *et al.* (1992 ▶).
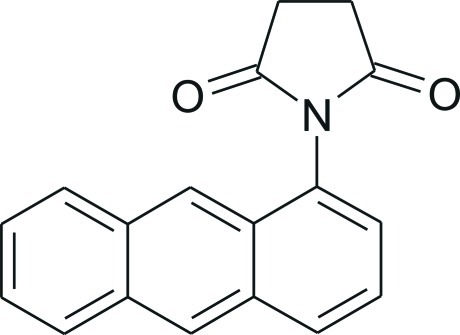


## Experimental

### 

#### Crystal data

C_18_H_13_NO_2_
*M* *_r_* = 275.29
Orthorhombic, 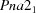

*a* = 18.4179 (9) Å
*b* = 5.7697 (4) Å
*c* = 12.4403 (6) Å
*V* = 1321.98 (13) Å^3^
*Z* = 4
Mo *K*α radiation
μ = 0.09 mm^−1^
*T* = 173 K
0.49 × 0.15 × 0.10 mm

#### Data collection

Bruker APEXII CCD diffractometer
10655 measured reflections
1667 independent reflections
1258 reflections with *I* > 2σ(*I*)
*R*_int_ = 0.081

#### Refinement

*R*[*F* ^2^ > 2σ(*F* ^2^)] = 0.041
*wR*(*F* ^2^) = 0.088
*S* = 0.95
1667 reflections
190 parameters
1 restraint
H-atom parameters constrained
Δρ_max_ = 0.17 e Å^−3^
Δρ_min_ = −0.19 e Å^−3^

### 

Data collection: *APEX2* (Bruker 2005 ▶); cell refinement: *SAINT* (Bruker 2005 ▶); data reduction: *SAINT*; program(s) used to solve structure: *SHELXS97* (Sheldrick, 2008 ▶); program(s) used to refine structure: *SHELXL97* (Sheldrick, 2008 ▶); molecular graphics: *ORTEP-3* (Farrugia, 1997 ▶) and *SCHAKAL99* (Keller, 1999 ▶); software used to prepare material for publication: *WinGX* (Farrugia, 1999 ▶) and *PLATON* (Spek, 2009 ▶).

## Supplementary Material

Crystal structure: contains datablock(s) global, I. DOI: 10.1107/S160053681201536X/rk2341sup1.cif

Structure factors: contains datablock(s) I. DOI: 10.1107/S160053681201536X/rk2341Isup2.hkl

Supplementary material file. DOI: 10.1107/S160053681201536X/rk2341Isup3.cml

Additional supplementary materials:  crystallographic information; 3D view; checkCIF report

## Figures and Tables

**Table 1:** Hydrogen-bond geometry (Å, °) *Cg*2 is the centroid of the C7–C12 ring.

*D*—H⋯*A*	*D*—H	H⋯*A*	*D*⋯*A*	*D*—H⋯*A*
C2—H2⋯O2^i^	0.95	2.38	3.234 (3)	149
C6—H6⋯O1^ii^	0.95	2.49	3.357 (3)	152
C9—H9⋯O2^iii^	0.95	2.53	3.465 (3)	167
C13—H13⋯O1^iv^	0.95	2.70	3.471 (3)	139
C17—H17*A*⋯*Cg*2^v^	0.99	2.92	3.709 (3)	138

Symmetry codes: (i) 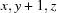; (ii) 
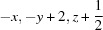
; (iii) 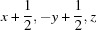; (iv) 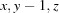; (v) 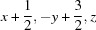.

**Fig. 1.**
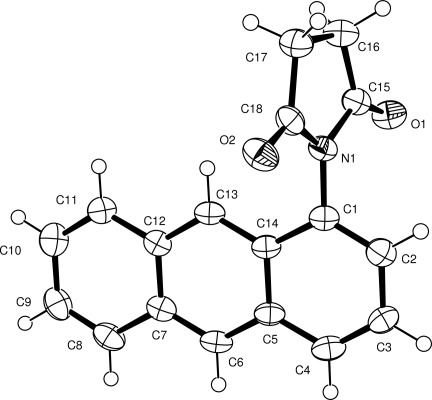
The molecular structure of the title compound, showing the atomic numbering scheme. Displacement ellipsoids are drawn at the 50% probability level. H atoms are presented as small spheres of arbitrary radius.

**Fig. 2.**
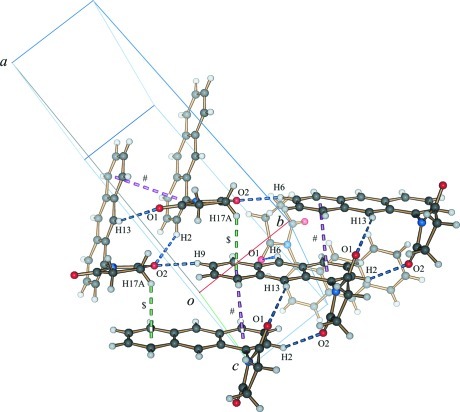
C–H···O, C–H···π and π–π interactions in the structure of the title compound. The C–H···π and π–π interactions are respectively indicated by dollar ($) or hash (#) symbols.

## supplementary crystallographic information

### Comment

The compound anthracene has been known for a long time and its properties have been extensively studied. The regio- and sterio-selectivity of substituted anthracenes in Diels-Alder reactions have been investigated and reported (Alston *et al.*, 1979; Meek *et al.*, 1960; Kaplan & Conroy, 1963; Verma & Singh, 1977; Singh & Ningombom, 2010). A study of the title compound and 1-succinimidonaphthalene involving synthesis, NMR experiments and molecular mechanics has been reported by Hubbard *et al.* (1992).

Both the anthracene and succinimide moeities are planar but are tilted with respect to each other at an angle of 71.94 (4)° (Fig. 1). Two anthracene bond lengths – C1—C14 [1.430 (3)Å] and C5—C14 [1.443 (3)Å] – are significantly longer than the 1.39Å typical of aromatic rings. As a consequence the rings containing these have been flagged as having larger than average C6-ring C—C bond lengths by *PLATON* (Spek, 2009), suggesting that the succinimide group has a significant effect on the charge distribution within the anthracene ring. The crystal structure contains three different types of intermolecular interactions, these include C–H···O, C–H···π and π–π interactions (Fig. 2). The π–π interaction occurs over a *Cg*1···*Cg*2 distance of 3.678 (2)Å between the rings defined by C1-C5/C14 (*Cg*1) and C7-C12 (*Cg*2). This leads to the stacking of molecules along *b* axis. Geometrical details of the C–H···π and C–H···O interactions are given in the Table 1.

### Experimental

The title compound was synthesized with very low yield (a few crystals) by reaction of 1-aminoanthracene (0.200 g, 1 mmol) with succinic anhydride (0.107 g, 1 mmol) in the presence of dioxane as a solvent (3 ml) by strirring at room temperature for a few hours. Thionyl chloride (3 ml) in dioxane (2 ml) was then slowly added to the reaction mixture at room temperature. The mixture was then kept at 323 K for 12 h, followed by neutralization of excess thionyl chloride by pouring the mixture into a beaker containing ice. This mixture was then filtered yielding a dark brown material, which after recrystallization by slow evaporation from chloroform yielded a few crystals suitable for analysis by X-ray diffraction.

### Refinement

All H atoms were positioned geometrically, and allowed to ride on their parent atoms, with C–H bond lengths of 0.95Å for aromatic H or 0.99Å for methylene H and *U*_iso_(H) = 1.2*U*_eq_(C). The 1449 Friedel pairs were merged during structure refinement.

### Crystal data

**Table:**

C_18_H_13_NO_2_	*F*(000) = 576
*M**_r_* = 275.29	*D*_x_ = 1.383 Mg m^−^^3^
Orthorhombic, *P**n**a*2_1_	Mo *K*α radiation, λ = 0.71073 Å
Hall symbol: P 2c -2n	Cell parameters from 2466 reflections
*a* = 18.4179 (9) Å	θ = 2.8–26.7°
*b* = 5.7697 (4) Å	µ = 0.09 mm^−^^1^
*c* = 12.4403 (6) Å	*T* = 173 K
*V* = 1321.98 (13) Å^3^	Plate, brown
*Z* = 4	0.49 × 0.15 × 0.10 mm

### Data collection

**Table:**

Bruker APEXII CCD diffractometer	1258 reflections with *I* > 2σ(*I*)
Radiation source: fine-focus sealed tube	*R*_int_ = 0.081
Graphite monochromator	θ_max_ = 28.0°, θ_min_ = 2.2°
φ– and ω–scans	*h* = −24→24
10655 measured reflections	*k* = −7→7
1667 independent reflections	*l* = −16→16

### Refinement

**Table:**

Refinement on *F*^2^	Primary atom site location: structure-invariant direct methods
Least-squares matrix: full	Secondary atom site location: difference Fourier map
*R*[*F*^2^ > 2σ(*F*^2^)] = 0.041	Hydrogen site location: inferred from neighbouring sites
*wR*(*F*^2^) = 0.088	H-atom parameters constrained
*S* = 0.95	*w* = 1/[σ^2^(*F*_o_^2^) + (0.0468*P*)^2^] where *P* = (*F*_o_^2^ + 2*F*_c_^2^)/3
1667 reflections	(Δ/σ)_max_ = 0.002
190 parameters	Δρ_max_ = 0.17 e Å^−^^3^
1 restraint	Δρ_min_ = −0.19 e Å^−^^3^

### Special details

**Table:**

Geometry. All s.u.'s (except s.u. in the dihedral angle between two l.s. planes) are estimated using the full covariance matrix. The cell s.u.'s are taken into account individually in the estimation of s.u.'s in distances, angles and torsion angles; correlations between s.u.'s in cell parameters are only used when they are defined by crystal symmetry. An approximate (isotropic) treatment of cell s.u.'s is used for estimating s.u.'s involving l.s. planes.
Refinement. Refinement of *F*^2^ against ALL reflections. The weighted *R*-factor *wR* and goodness of fit *S* are based on *F*^2^, conventional *R*-factors *R* are based on *F*, with *F* set to zero for negative *F*^2^. The threshold expression of *F*^2^ > σ(*F*^2^) is used only for calculating *R*-factors(gt) *etc*. and is not relevant to the choice of reflections for refinement. *R*-factors based on *F*^2^ are statistically about twice as large as those based on *F*, and *R*-factors based on ALL data will be even larger.

### Fractional atomic coordinates and isotropic or equivalent isotropic displacement parameters (Å^2^)

**Table:**

	*x*	*y*	*z*	*U*_iso_*/*U*_eq_	
C1	−0.12512 (12)	1.0166 (5)	0.33781 (19)	0.0300 (6)	
C2	−0.13405 (14)	1.2022 (5)	0.40282 (19)	0.0348 (7)	
H2	−0.1762	1.2958	0.3959	0.042*	
C3	−0.08086 (14)	1.2588 (5)	0.4816 (2)	0.0369 (7)	
H3	−0.0870	1.3914	0.5259	0.044*	
C4	−0.02145 (13)	1.1223 (5)	0.4929 (2)	0.0373 (7)	
H4	0.0131	1.1585	0.5471	0.045*	
C5	−0.00952 (13)	0.9262 (5)	0.42571 (18)	0.0313 (6)	
C6	0.05236 (13)	0.7881 (5)	0.43475 (19)	0.0340 (7)	
H6	0.0869	0.8231	0.4891	0.041*	
C7	0.06517 (13)	0.6010 (5)	0.3669 (2)	0.0331 (6)	
C8	0.12738 (13)	0.4548 (5)	0.3779 (2)	0.0393 (7)	
H8	0.1618	0.4860	0.4329	0.047*	
C9	0.13797 (14)	0.2722 (6)	0.3111 (2)	0.0420 (7)	
H9	0.1795	0.1764	0.3200	0.050*	
C10	0.08711 (14)	0.2228 (6)	0.2274 (2)	0.0429 (7)	
H10	0.0952	0.0961	0.1801	0.051*	
C11	0.02709 (13)	0.3575 (5)	0.2156 (2)	0.0351 (7)	
H11	−0.0065	0.3229	0.1599	0.042*	
C12	0.01350 (12)	0.5483 (5)	0.28448 (18)	0.0300 (6)	
C13	−0.04932 (12)	0.6848 (5)	0.27515 (19)	0.0298 (6)	
H13	−0.0837	0.6496	0.2206	0.036*	
C14	−0.06241 (12)	0.8710 (5)	0.34406 (18)	0.0280 (6)	
C15	−0.19237 (13)	1.1049 (5)	0.16742 (19)	0.0324 (6)	
C16	−0.24985 (14)	0.9892 (5)	0.1008 (2)	0.0409 (7)	
H16A	−0.2302	0.9440	0.0298	0.049*	
H16B	−0.2917	1.0943	0.0898	0.049*	
C17	−0.27266 (14)	0.7749 (5)	0.1650 (2)	0.0394 (7)	
H17A	−0.3250	0.7813	0.1828	0.047*	
H17B	−0.2630	0.6316	0.1236	0.047*	
C18	−0.22734 (12)	0.7812 (5)	0.2656 (2)	0.0328 (6)	
N1	−0.17925 (10)	0.9652 (4)	0.25776 (16)	0.0301 (5)	
O1	−0.16111 (10)	1.2847 (4)	0.14866 (14)	0.0409 (5)	
O2	−0.23048 (9)	0.6519 (4)	0.34224 (15)	0.0415 (5)	

### Atomic displacement parameters (Å^2^)

**Table:**

	*U*^11^	*U*^22^	*U*^33^	*U*^12^	*U*^13^	*U*^23^
C1	0.0272 (12)	0.0376 (16)	0.0252 (11)	−0.0043 (11)	0.0015 (9)	0.0022 (13)
C2	0.0324 (13)	0.0423 (18)	0.0298 (13)	0.0020 (12)	0.0038 (10)	0.0024 (14)
C3	0.0414 (15)	0.0417 (18)	0.0278 (12)	−0.0020 (13)	0.0027 (11)	−0.0064 (13)
C4	0.0360 (14)	0.051 (2)	0.0253 (12)	−0.0075 (13)	0.0000 (11)	−0.0014 (14)
C5	0.0294 (12)	0.0421 (17)	0.0223 (11)	−0.0043 (12)	0.0000 (10)	0.0022 (12)
C6	0.0275 (12)	0.0484 (19)	0.0260 (12)	−0.0051 (12)	−0.0050 (10)	0.0036 (14)
C7	0.0280 (12)	0.0402 (18)	0.0312 (13)	−0.0038 (12)	0.0001 (10)	0.0069 (13)
C8	0.0262 (12)	0.050 (2)	0.0419 (14)	−0.0011 (13)	−0.0026 (11)	0.0111 (15)
C9	0.0290 (13)	0.0429 (19)	0.0541 (17)	0.0058 (13)	0.0047 (13)	0.0080 (16)
C10	0.0365 (15)	0.0420 (19)	0.0503 (17)	0.0007 (14)	0.0099 (13)	−0.0003 (17)
C11	0.0320 (14)	0.0402 (18)	0.0333 (13)	−0.0010 (13)	0.0013 (10)	−0.0016 (13)
C12	0.0260 (12)	0.0353 (17)	0.0287 (12)	−0.0020 (11)	0.0030 (9)	0.0051 (13)
C13	0.0258 (11)	0.0403 (18)	0.0235 (11)	−0.0061 (11)	0.0000 (9)	0.0032 (13)
C14	0.0261 (11)	0.0361 (16)	0.0217 (11)	−0.0054 (11)	0.0019 (9)	0.0022 (12)
C15	0.0279 (12)	0.0426 (17)	0.0267 (12)	0.0034 (12)	0.0039 (9)	0.0004 (13)
C16	0.0381 (13)	0.052 (2)	0.0326 (12)	−0.0054 (14)	−0.0045 (10)	0.0022 (14)
C17	0.0344 (13)	0.0445 (18)	0.0393 (14)	−0.0035 (12)	−0.0058 (11)	0.0011 (14)
C18	0.0260 (12)	0.0367 (16)	0.0357 (13)	0.0043 (11)	0.0009 (10)	0.0031 (14)
N1	0.0257 (9)	0.0377 (13)	0.0269 (9)	0.0010 (9)	−0.0007 (8)	0.0015 (10)
O1	0.0431 (10)	0.0482 (13)	0.0313 (9)	−0.0103 (10)	0.0025 (8)	0.0046 (9)
O2	0.0349 (9)	0.0453 (12)	0.0443 (11)	−0.0037 (9)	−0.0026 (8)	0.0134 (10)

### Geometric parameters (Å, º)

**Table:**

C1—C2	1.352 (4)	C10—C11	1.359 (4)
C1—C14	1.430 (3)	C10—H10	0.9500
C1—N1	1.440 (3)	C11—C12	1.418 (4)
C2—C3	1.423 (4)	C11—H11	0.9500
C2—H2	0.9500	C12—C13	1.405 (3)
C3—C4	1.355 (4)	C13—C14	1.395 (3)
C3—H3	0.9500	C13—H13	0.9500
C4—C5	1.424 (4)	C15—O1	1.209 (3)
C4—H4	0.9500	C15—N1	1.404 (3)
C5—C6	1.395 (4)	C15—C16	1.501 (4)
C5—C14	1.443 (3)	C16—C17	1.531 (4)
C6—C7	1.390 (4)	C16—H16A	0.9900
C6—H6	0.9500	C16—H16B	0.9900
C7—C8	1.429 (4)	C17—C18	1.505 (3)
C7—C12	1.432 (3)	C17—H17A	0.9900
C8—C9	1.357 (4)	C17—H17B	0.9900
C8—H8	0.9500	C18—O2	1.211 (3)
C9—C10	1.430 (4)	C18—N1	1.386 (3)
C9—H9	0.9500		
			
C2—C1—C14	122.1 (2)	C12—C11—H11	119.3
C2—C1—N1	119.5 (2)	C13—C12—C11	122.1 (2)
C14—C1—N1	118.4 (2)	C13—C12—C7	119.2 (2)
C1—C2—C3	120.6 (3)	C11—C12—C7	118.7 (2)
C1—C2—H2	119.7	C14—C13—C12	121.6 (2)
C3—C2—H2	119.7	C14—C13—H13	119.2
C4—C3—C2	119.6 (3)	C12—C13—H13	119.2
C4—C3—H3	120.2	C13—C14—C1	123.9 (2)
C2—C3—H3	120.2	C13—C14—C5	119.1 (2)
C3—C4—C5	121.7 (2)	C1—C14—C5	117.0 (2)
C3—C4—H4	119.2	O1—C15—N1	124.4 (2)
C5—C4—H4	119.2	O1—C15—C16	127.7 (2)
C6—C5—C4	122.2 (2)	N1—C15—C16	107.9 (2)
C6—C5—C14	118.8 (2)	C15—C16—C17	105.4 (2)
C4—C5—C14	119.0 (2)	C15—C16—H16A	110.7
C7—C6—C5	122.2 (2)	C17—C16—H16A	110.7
C7—C6—H6	118.9	C15—C16—H16B	110.7
C5—C6—H6	118.9	C17—C16—H16B	110.7
C6—C7—C8	122.4 (2)	H16A—C16—H16B	108.8
C6—C7—C12	119.1 (2)	C18—C17—C16	105.2 (2)
C8—C7—C12	118.4 (3)	C18—C17—H17A	110.7
C9—C8—C7	121.0 (3)	C16—C17—H17A	110.7
C9—C8—H8	119.5	C18—C17—H17B	110.7
C7—C8—H8	119.5	C16—C17—H17B	110.7
C8—C9—C10	120.5 (3)	H17A—C17—H17B	108.8
C8—C9—H9	119.8	O2—C18—N1	123.9 (2)
C10—C9—H9	119.8	O2—C18—C17	127.8 (2)
C11—C10—C9	119.8 (3)	N1—C18—C17	108.3 (2)
C11—C10—H10	120.1	C18—N1—C15	112.7 (2)
C9—C10—H10	120.1	C18—N1—C1	123.5 (2)
C10—C11—C12	121.5 (3)	C15—N1—C1	123.7 (2)
C10—C11—H11	119.3		
			
C14—C1—C2—C3	−0.6 (4)	C2—C1—C14—C13	−176.7 (2)
N1—C1—C2—C3	−179.1 (2)	N1—C1—C14—C13	1.8 (3)
C1—C2—C3—C4	−1.4 (4)	C2—C1—C14—C5	1.9 (3)
C2—C3—C4—C5	1.9 (4)	N1—C1—C14—C5	−179.5 (2)
C3—C4—C5—C6	178.3 (3)	C6—C5—C14—C13	−1.4 (3)
C3—C4—C5—C14	−0.4 (4)	C4—C5—C14—C13	177.3 (2)
C4—C5—C6—C7	−177.9 (2)	C6—C5—C14—C1	179.8 (2)
C14—C5—C6—C7	0.8 (4)	C4—C5—C14—C1	−1.4 (3)
C5—C6—C7—C8	−178.1 (3)	O1—C15—C16—C17	−177.3 (2)
C5—C6—C7—C12	0.4 (4)	N1—C15—C16—C17	3.5 (3)
C6—C7—C8—C9	179.4 (3)	C15—C16—C17—C18	0.6 (3)
C12—C7—C8—C9	0.9 (4)	C16—C17—C18—O2	175.4 (3)
C7—C8—C9—C10	0.4 (4)	C16—C17—C18—N1	−4.5 (3)
C8—C9—C10—C11	−1.0 (4)	O2—C18—N1—C15	−172.7 (2)
C9—C10—C11—C12	0.3 (4)	C17—C18—N1—C15	7.2 (3)
C10—C11—C12—C13	−178.2 (3)	O2—C18—N1—C1	3.1 (4)
C10—C11—C12—C7	1.1 (4)	C17—C18—N1—C1	−177.0 (2)
C6—C7—C12—C13	−0.9 (3)	O1—C15—N1—C18	173.9 (2)
C8—C7—C12—C13	177.6 (2)	C16—C15—N1—C18	−6.9 (3)
C6—C7—C12—C11	179.9 (2)	O1—C15—N1—C1	−1.8 (4)
C8—C7—C12—C11	−1.6 (4)	C16—C15—N1—C1	177.4 (2)
C11—C12—C13—C14	179.4 (2)	C2—C1—N1—C18	−107.2 (3)
C7—C12—C13—C14	0.2 (3)	C14—C1—N1—C18	74.2 (3)
C12—C13—C14—C1	179.6 (2)	C2—C1—N1—C15	68.1 (3)
C12—C13—C14—C5	0.9 (3)	C14—C1—N1—C15	−110.5 (3)

### Hydrogen-bond geometry (Å, º)

Cg2 is the centroid of the C7–C12 ring.

**Table:**

*D*—H···*A*	*D*—H	H···*A*	*D*···*A*	*D*—H···*A*
C2—H2···O2^i^	0.95	2.38	3.234 (3)	149
C6—H6···O1^ii^	0.95	2.49	3.357 (3)	152
C9—H9···O2^iii^	0.95	2.53	3.465 (3)	167
C13—H13···O1^iv^	0.95	2.70	3.471 (3)	139
C17—H17*A*···*Cg*2^v^	0.99	2.92	3.709 (3)	138

Symmetry codes: (i) *x*, *y*+1, *z*; (ii) −*x*, −*y*+2, *z*+1/2; (iii) *x*+1/2, −*y*+1/2, *z*; (iv) *x*, *y*−1, *z*; (v) *x*+1/2, −*y*+3/2, *z*.
